# Supplementary material for: Linking individual differences in human primary visual cortex to contrast sensitivity around the visual field
Source: Nat Commun. 2022 Jun 13;13:3309. doi: 10.1038/s41467-022-31041-9 (PMC9192713; doi:10.1038/s41467-022-31041-9)
Supplement: Supplementary file 3 — Reporting Summary [file 41467_2022_31041_MOESM3_ESM.pdf]

nature portfolio

Corresponding author(s): Marc Himmelberg

Last updated by author(s): Apr 13, 2022

Reporting Summary

Nature Portfolio wishes to improve the reproducibility of the work that we publish. This form provides structure for consistency and transparency in reporting. For further information on Nature Portfolio policies, see our Editorial Policies and the Editorial Policy Checklist.

Statistics

For all statistical analyses, confirm that the following items are present in the figure legend, table legend, main text, or Methods section.

n/a	Confirmed	
☐	☒	The exact sample size (*n*) for each experimental group/condition, given as a discrete number and unit of measurement
☐	☒	A statement on whether measurements were taken from distinct samples or whether the same sample was measured repeatedly
☐	☒	The statistical test(s) used AND whether they are one- or two-sided<br>*Only common tests should be described solely by name; describe more complex techniques in the Methods section.*
☒	☐	A description of all covariates tested
☒	☐	A description of any assumptions or corrections, such as tests of normality and adjustment for multiple comparisons
☐	☒	A full description of the statistical parameters including central tendency (e.g. means) or other basic estimates (e.g. regression coefficient) AND variation (e.g. standard deviation) or associated estimates of uncertainty (e.g. confidence intervals)
☐	☒	For null hypothesis testing, the test statistic (e.g. *F*, *t*, *r*) with confidence intervals, effect sizes, degrees of freedom and *P* value noted<br>*Give P values as exact values whenever suitable.*
☒	☐	For Bayesian analysis, information on the choice of priors and Markov chain Monte Carlo settings
☒	☐	For hierarchical and complex designs, identification of the appropriate level for tests and full reporting of outcomes
☐	☒	Estimates of effect sizes (e.g. Cohen's *d*, Pearson's *r*), indicating how they were calculated

Our web collection on statistics for biologists contains articles on many of the points above.

Software and code

Policy information about availability of computer code

Data collection	fMRI stimuli were presented using MATLAB 2017A, Psychtoolbox V3 (http://psychtoolbox.org/), and the vistadisp toolbox (https://github.com/WinawerLab/vistadisp). Psychophysics stimuli were generated using MATLAB 2017a using the MGL Toolbox V2.0 (http://gru.stanford.edu/doku.php/mgl/overview).
Data analysis	fMRI data were preprocessed using fMRIprep v20.0.1 and additional analyses were completed using custom MATLAB code, the Neuropythy V0.11.9 python package (https://github.com/noahbenson/neuropythy), and a customised version of the vistasoft toolbox (https://vistalab.stanford.edu/software/) . Psychophysics data were analysed using custom MATLAB code. Eyetracking data were analysed with the Eyelink Toolbox V3.

For manuscripts utilizing custom algorithms or software that are central to the research but not yet described in published literature, software must be made available to editors and reviewers. We strongly encourage code deposition in a community repository (e.g. GitHub). See the Nature Portfolio guidelines for submitting code & software for further information.

Data

Policy information about availability of data

All manuscripts must include a data availability statement. This statement should provide the following information, where applicable:

- Accession codes, unique identifiers, or web links for publicly available datasets
- A description of any restrictions on data availability
- For clinical datasets or third party data, please ensure that the statement adheres to our policy

Source data are provided with this paper. The data generated for this study have been deposited in the OSF repository https://osf.io/de7zg.

Field-specific reporting

Please select the one below that is the best fit for your research. If you are not sure, read the appropriate sections before making your selection.

☐ Life sciences ☒ Behavioural & social sciences ☐ Ecological, evolutionary & environmental sciences

For a reference copy of the document with all sections, see nature.com/documents/nr-reporting-summary-flat.pdf

Behavioural & social sciences study design

All studies must disclose on these points even when the disclosure is negative.

Study description	Quantitative repeated measures study; correlation
Research sample	Members of the New York University community (undergraduates, masters students, PhD students, post-docs, and faculty) 18 females, 11 males, mean age = 29.9 years. All participants had normal vision and no history of neurological or psychiatric illness. The study assessed human vision which does not greatly vary key characteristics (i.e., age, gender, socioeconomic status, etc) thus this sample is representative typical vision across the the population and is considered an appropriate study sample.
Sampling strategy	We sampled from the participant pool at convenience and availability without regard to age, gender, or handedness. We aimed for 30 participants based on the sample size of prior studies conducting correlations of fMRI and behavioral measurements of vision (https://doi.org/10.1038/<br>nn .2706, https://doi .org/10.1038/ncomms3201, http://dx.doi.org/10.1016/j. neu ron.2014.12.041).
Data collection	Psychophysical stimuli were presented on a 21-inch Viewsonic G220fb CRT monitor (1280 x 960 resolution, 100 Hz) and were generated using an Apple iMac (3.2 GHz, Intel Core i3) in MATLAB 2017a (Mathworks, Natick, MA). Keyboard responses were recorded using MGL Toolbox running via MATLAB 2017a.<br>Anatomical and functional MRI data were acquired on a 3T Siemens MAGNETOM Prisma MRI scanner (Siemens Medical Solutions, Erlangen, Germany) using a Siemens 64-channel head coil.<br><br>During data collection, nobody else was present other than the participant and the researcher. Two authors (MMH and JW) participated in the study were not blinded to the study hypothesis. All other participants were blinded to the research hypotheses during data collection.
Timing	Data were collected between 3rd November 2019 and 21 July 2021
Data exclusions	No data were excluded from the analysis
Non-participation	No participants declined or dropped out of the study
Randomization	Participants were not randomized into experimental groups - the repeated measures design meant they participated in both experiments. Controlling of covariates is not relevant to this work; any covariates among our sample do not have influence on human vision and would not affect the outcome of this experiment.

Reporting for specific materials, systems and methods

We require information from authors about some types of materials, experimental systems and methods used in many studies. Here, indicate whether each material, system or method listed is relevant to your study. If you are not sure if a list item applies to your research, read the appropriate section before selecting a response.

Materials & experimental systems

n/a	Involved in the study	
☒	☐	Antibodies
☒	☐	Eukaryotic cell lines
☒	☐	Palaeontology and archaeology
☒	☐	Animals and other organisms
☐	☒	Human research participants
☒	☐	Clinical data
☒	☐	Dual use research of concern

Methods

n/a	Involved in the study	
☒	☐	ChIP-seq
☒	☐	Flow cytometry
☐	☒	MRI-based neuroimaging

Human research participants

Policy information about studies involving human research participants

Population characteristics	See above

Recruitment	Participants were recruited in person based on availability. Participants ranged from experienced psychophysical observers to those who had never participated in a psychophysics or fMRI study before. Regardless, experience should make no impact to our data or its interpretation. All participants (other than two authors) were naive to the purpose of the study. Participants were paid for their participation in the experiment however this would not create a bias or impact results as they were naive to the purpose of the study and these factors do not effect the biological measures made here.
Ethics oversight	The experiment was conducted in accordance with the Declaration of Helsinki and was approved by the New York University ethics committee on activities involving human observers.

Note that full information on the approval of the study protocol must also be provided in the manuscript.

Magnetic resonance imaging

Experimental design

Design type	Population receptive field mapping
Design specifications	Each participant completed 1 session that consisted of 4 - 12 fMRI scans. The number of scans differed based on time available in the scan session. Each scan lasted 3.5 minutes.
Behavioral performance measures	Button press; participants pressed a button when fixation cross changed colour. The task is used to ensure that the participant is awake during the task and maintaining fixation. The % correct identification of the fixation cross changing colour was used to assess that the participant was awake and fixation and is of otherwise no relevance to the study.

Acquisition

Imaging type(s)	Anatomical MRI and functional MRI
Field strength	3T
Sequence & imaging parameters	Functional echo-planar images (EPIs) were acquired for each observer using a T2*-weighted multiband EPI sequence (TR, 1000 ms; TE, 37 ms; voxel size, 2mm3; flip angle, 68° ; multiband acceleration factor, 6; phase-encoding, posterioranterior)
Area of acquisition	Whole brain (Seimens 64-channel head coil)

Diffusion MRI ☐ Used ☒ Not used

Preprocessing

Preprocessing software	fmriPrep v.20.0.1
Normalization	Data were not normalized but were rather analyzed on each participant's native brain surface (fsnative surface) as the study intended to assess individual differences, thus normalization to a standard space was not appropriate.
Normalization template	The data were not normalized
Noise and artifact removal	The pRF analysis detrends the data by projecting out 3 low frequency vectors (the first 3 terms of the discrete cosine transform). There is no other de-noising or artifact removal. Repeated scans with identical stimuli combined with a model based analysis make the paradigm robust to noise
Volume censoring	Data were not censored as there were minimal artifacts; for each participant, their scans and the volumes within were averaged and included as input into the fMRI population receptive field model.

Statistical modeling & inference

Model type and settings	Retinotopic maps were generated using the population receptive field model
Effect(s) tested	Fit of the pRF model to the time-series data (vertex-wise)

Specify type of analysis: ☒ Whole brain ☐ ROI-based ☐ Both

Statistic type for inference (See Eklund et al. 2016)	Population receptive field model was fit to each vertex on the whole brain surface (vertex-wise). We did not do null hypothesis testing of the fMRI data alone.
Correction	Not appropriate for the population receptive field model.

Models & analysis

n/a	Involved in the study
☒	☐ Functional and/or effective connectivity
☒	☐ Graph analysis
☒	☐ Multivariate modeling or predictive analysis
